# Non-Invasive Assessment of Multivalvular Heart Disease: A Comprehensive Review

**DOI:** 10.31083/j.rcm2501029

**Published:** 2024-01-16

**Authors:** Giulia De Zan, Ivo A. C. van der Bilt, Lysette N. Broekhuizen, Maarten J. Cramer, Ibrahim Danad, Dirk van Osch, Giuseppe Patti, Philippe J. van Rosendael, Arco J. Teske, Pim van der Harst, Marco Guglielmo

**Affiliations:** ^1^Department of Translational Medicine, Division of Cardiology, University of Eastern Piedmont, Maggiore della Carità Hospital, 28100 Novara, Italy; ^2^Department of Cardiology, Division of Heart and Lungs, Utrecht University Medical Center, 3584 CX Utrecht, The Netherlands; ^3^Department of Cardiology, Haga Teaching Hospital, 2545 AA The Hague, The Netherlands

**Keywords:** multivalvular heart disease, cardiovascular imaging, echocardiography

## Abstract

Multivalvular heart disease (MVD) implies the presence of concomitant valvular lesions on two or more heart valves. This condition has become common in the few last years, mostly due to population aging. Every combination of valvular lesions uniquely redefines the hemodynamics of a patient. Over time, this may lead to alterations in left ventricle (LV) dimensions, shape and, eventually, function. Since most of the echocardiographic parameters routinely used in the valvular assessment have been developed in the context of single valve disease and are frequently flow- and load-dependent, their indiscriminate use in the context of MVD can potentially lead to errors in judging lesion severity. Moreover, the combination of non-severe lesions may still cause severe hemodynamic consequences, and thereby systolic dysfunction. This review aims to discuss the most frequent combinations of MVD and their echocardiographic caveats, while addressing the opportunities for a multimodality assessment to achieve a better understanding and treatment of these patients.

## 1. Introduction

Multivalvular heart disease (MVD) is defined as the presence of combined stenotic or regurgitant lesions occurring on more than one heart valve [1].

It is estimated that over 30% of patients older than 65 years have MVD, defining this condition as rather common among the aging population [2]. The EuroHeart Survey outlined that one out of five patients with valvular heart disease (VHD), and 14.6% of those receiving valvular surgery, had MVD [3]. In the same registry, patients with MVD had a mean age of 64 ± 14 years. In the more recent EURObservational Research Programme Valvular Heart Disease II Survey that included patients with at least one severe lesion, those with MVD accounted for up to 27.8% of the overall population, and were more frequently women affected by chronic kidney failure and atrial fibrillation. The most frequent combination was the presence of severe aortic stenosis (AS) and moderate mitral regurgitation (MR) [4]. These data are consistent with the results of the PARTNER 2 trial, which also showed the presence of a coexisting significant MR and tricuspid regurgitation (TR) in 20% and 27% of patients receiving transcatheter aortic valve replacement (TAVR), respectively [5]. According to the results of the PARTNER trials, it seems that the higher the risk of the patient with AS, the higher the incidence of concomitant MR. While in high-risk and inoperable patients, the prevalence of at least moderate MR was of 21% and 23% respectively, in low-risk patients it did not exceed 3% [6, 7]. Similarly, more than 10% of the valve surgeries in the database of the Society of Thoracic Surgeons were indeed surgeries on more than one cardiac valve, with more than half of them involving a combination of aortic and mitral valve lesions [8].

In the same registry, rheumatic heart disease appears as the main cause (51%) of MVD, and the second was of degenerative etiology (41%) [8]. Endocarditis, iatrogenic causes such as radiotherapy and adverse drug effects, connective tissue diseases and congenital valvular diseases. As for secondary MVD, the co-existence of MR and TR is typically secondary to leaflets malcoaptation due to alterations in the geometry of the ventricles or atria. Moreover, primary and secondary aetiologies can coexist: in a review by Nombela-Franco *et al*. [9], secondary MR accounted for half of the patients with MR undergoing TAVR.

The wide range of possible pathophysiological combinations leads to different clinical scenarios and makes MVD a complex phenomenon to study. Echocardiography is the main technique for diagnosing aetiology, severity and often guides the decision for intervention. The main setback is that the well-validated cut-off values are suited for single valvular disease and are not easy to apply in MVD, most often due to hemodynamic changes in the ventricles. As a result, existing data on MVD are limited despite its prevalence and the management of these patients is not thoroughly covered by current guidelines, with indications mainly based on small studies or consensus opinions [10, 11].

In this review, we will display the most frequent MVD combinations and their echocardiographic pitfalls, thus addressing the opportunities for a multimodality assessment of these patients.

## 2. Pathophysiological Considerations

The hemodynamic consequences of MVD influence ventricular size, shape, function and, eventually, the resulting clinical signs and symptoms. More specifically, changes in hemodynamics depend on the severity of the singular lesions, the combination of valvular diseases at play, the aetiology (primary or secondary) and the chronicity of the lesions [12].

**Fig. 1.**
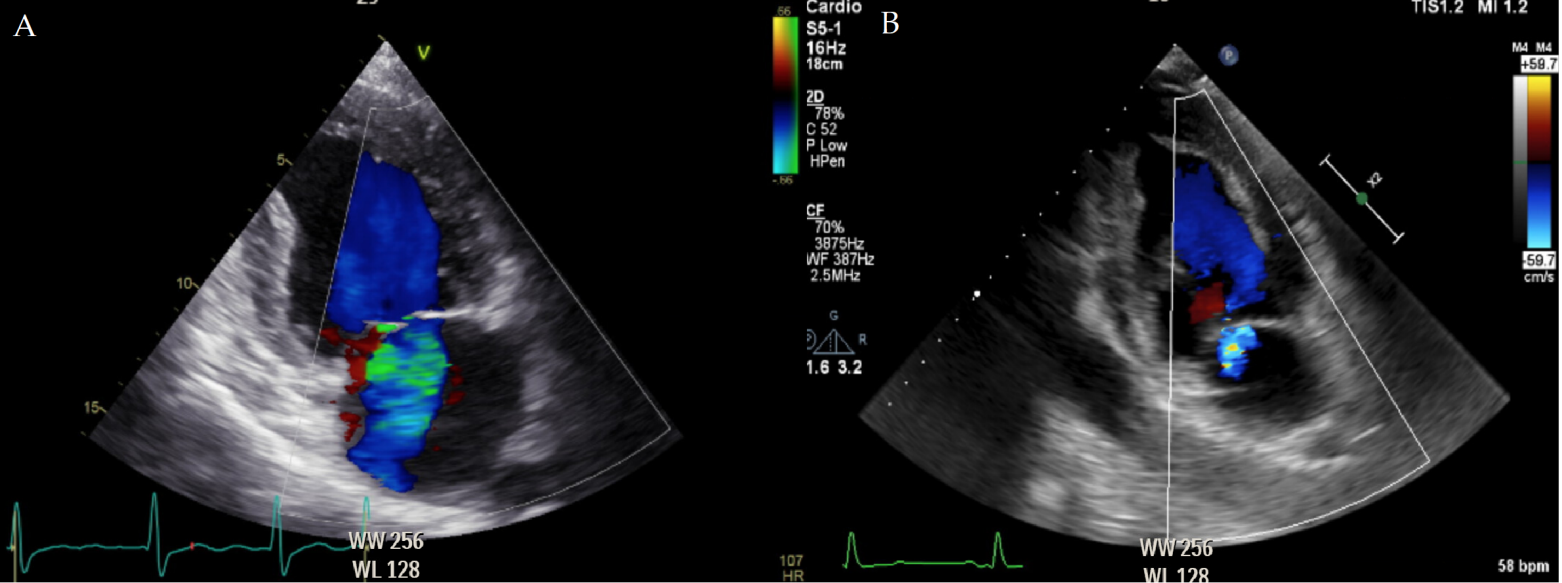
**Mitral regurgitation prior and after correction of aortic stenosis**. (A) A patient waiting for transcatheter aortic valve replacement undergoes an echocardiogram, which shows the concomitant presence of moderate mitral regurgitation, secondary to the pressure overload due to severe aortic stenosis. (B) The echocardiogram performed 24 hours after the aortic stenosis correction displays just a trace of mitral regurgitation, as a consequence of the normalization of left ventricular pressures. Later on, left ventricular remodeling can potentially further contribute to the reduction of mitral regurgitation severity.

The interplay between different valve lesions can either enhance or blur the hemodynamic effect of the single lesions. For instance, a patient with significant mitral stenosis (MS) and aortic regurgitation (AR) might develop left ventricle (LV) dilation later, due to the possible protection given by MS from the volume overload [13]. This hemodynamic interdependence is well known in the case of treatment of one of the valve lesions directly impacting the severity of the concomitant one. In several patients, an improvement in MR severity is common following treatment of AS, irrespective of the used technique (Fig. 1) [14, 15, 16].

## 3. Echocardiographic Assessment

As already mentioned, echocardiography is the main tool for the diagnosis of VHD. Evaluation of valve anatomy and dysfunction and quantification of stenosis or regurgitation in particular should be the result of a multiparametric analysis [17, 18]. Furthermore, it is important to evaluate both the left and right ventricular size, systolic and diastolic function, and a possible increase in pulmonary pressure. However, it is worth noting that various methods commonly employed to assess the severity of valve lesions have been validated only in the setting of single VHD. As a result, their reliability in the context of MVD may be limited by the hemodynamic changes discussed in Section 2. In general, it is preferable to rely on measurements that are less dependent on the patient’s loading conditions, like direct planimetry for stenotic lesions or, in the case of regurgitant valves, the vena contracta or the effective regurgitant orifice area (EROA). For example, in the presence of at least moderate AR, it is not advisable to calculate the mitral valve area (MVA) with the continuity equation method, as the continuous transmitral and transaortic flow are not the same in this condition. Similarly, we should not rely on the values obtained by continuous wave transmitral Doppler recordings in these patients since the rapid increase in LV diastolic pressure directly affects the rate of mitral inflow. An overview of the most important diagnostic echocardiographic caveats and their possible overcoming in the setting of MVD is displayed in Table 1.

**Table 1.** **Warnings in the echocardiographic assessment of left-sided multivalvular heart disease**.

Valvular lesion	MR	MS
AR	PHT unreliable (rapid filling shortens AR PHT)	Continuity equation for MVA not reliable (different flows)
	Doppler method for volume quantification using left-sided forward flow not valid mitral-to-aortic VTI ratio not reliable	PHT not reliable (mitral PHT is shortened by significant AR)
	Solutions	Solutions
	PISA method still reliable for MR	2D or 3D echocardiography to measure anatomic MVA
	CMR to quantify aortic and mitral RV and RF	Using pulmonic flow for the continuity equation
AS	Increased mitral RV	Low MS and AS (more frequently) gradients can occur
	Big area of MR jet on color-flow	PHT for MS unreliable
	Low-flow, low-gradient AS not uncommon	
	Solutions	Solutions
	EROA usually less affected	3D echocardiography to measure anatomic MVA
	CMR to quantify mitral RV and RF	DSE or calcium scoring on CT for AS severity
	DSE or calcium scoring on CT for AS severity	VTI LVOT/VTI AV for low flow-low gradient AS due to the concomitant valvulopathy
	VTI LVOT/VTI AV for low flow-low gradient AS due to the concomitant valvulopathy	

The table displays all the possible left-sided valvular lesion combinations, focusing on the echocardiographic pitfalls encountered in the severity assessment of multivalvular heart disease. For every combination, some methods usually valid in case of single valve disease are to be avoided in MVD. Preferred and more reliable methods are listed. In some cases this might mean relying on different imaging techniques. 2D, two-dimensional; 3D, three-dimensional; AR, aortic regurgitation; AS, aortic stenosis; CMR, cardiac magnetic resonance; CT, computed tomography; DSE, dobutamine stress echocardiography; EROA, effective regurgitant orifice ares; MR, mitral regurgitation; MS, mitral stenosis; MVA, mitral valve aerea; PHT, pressure-half time; PISA, proximal isovelocity surface area; RF, regurgitant fraction; RV, regurgitant volume; VTI, velocity-time integral.

### 3.1 Mitral Stenosis and Aortic Stenosis

The coexistence of significant AS and MS is more typical of rheumatic disease but demographics vary between different regions of the world. For example, in Western countries the combination of MS and AS affects mostly the aging population and has a degenerative etiology [19]. The latter doesn’t imply commissural fusion, which usually results in less severe stenosis than in rheumatic disease [20, 21]. Other rarer aetiologies include iatrogenic (both drug-induced and post-radiotherapy) and genetic (such as mucopolysaccharidosis) conditions.

Echocardiography is usually enough to give a comprehensive diagnosis when classical high gradients are recorded on both valves. Nonetheless, the reduction in flow through a severe valve lesion can affect the gradients across the valve [22]*.* This is more common for the aortic valve, being the distal lesion, but low gradients despite severe stenosis can also affect the mitral valve since a significant AS creates a low-flow condition itself.

Similarly, LV diastolic dysfunction due to AS can lead either to an underestimation of MVA due to an increase of mitral E wave half-pressure time in patients with abnormal relaxation or to an overestimation of MVA in case of a restrictive filling pattern [23].

Therefore, in this situation the planimetric assessment or the continuity equation are preferred over other methods to determine the severity of AS and MS. Furthermore, the proximal isovelocity surface area (PISA) approach continues to be a valuable tool for assessing the MVA in individuals with bivalvular rheumatic disease, while still lacking formal validation in the context of degenerative mitral valve conditions [19].

### 3.2 Aortic Stenosis and Mitral Regurgitation

The MR-AS combination is the most prevalent MVD in developed countries [3]. Even though the increased afterload due to AS classically leads to a hypertrophic LV, a considerable numbers of individuals with AS proceed to LV dilation and systolic impairment. This can be related to the excessively high LV afterload, concomitant cardiomyopathy (especially ischemic), or both. LV dilation and adverse remodeling can consequently lead to secondary MR as a result of dilation of the annulus and tethering of the leaflets [24]. Généreux *et al*. [25], categorized patients with severe AS and waiting for intervention into five stages, depending on the presence of extravalvular (extra aortic valve) cardiac damage or dysfunction on transthoracic echocardiography. In particular, stage 2 included left atrium or mitral valve damage, which was a predictor of mortality in this cohort of patients. Moreover, in patients with AS, the presence of MR may preserve ejection fraction despite impending LV dysfunction.

Of course, even though less frequently, primary MR in patients with AS can coexist as well, with similar hemodynamic effects.

On echocardiography, the AS-related increase in the pressure gradient over the mitral valve during systole AS will cause an augmentation of the regurgitant volume (RV) for any given mitral EROA [26]. EROA itself is usually less affected in these cases, and therefore preferable to assess MR severity.

At the same time, a significant MR can interfere in the echocardiographic evaluation of patients with AS, similar to what is described for the coexistence of MS and AS in section 3.1. A significant MR leads to a decreased flow over the aortic valve, which results in low transaortic gradients despite a narrow aortic valve area (AVA). The role of dobutamine stress echocardiography in this setting is still unclear, since data are lacking and both improvement and worsening of MR after dobutamine administration have been reported [27, 28]. Therefore, the effect of dobutamine administration on patients’ flow status in the case of concomitant AS and MR is not completely predictable. However, incase the needed increase in flow is achieved, dobutamine stress echocardiography can still be used to distinguish between true-severe and pseudo-severe AS.

### 3.3 Aortic Regurgitation and Mitral Regurgitation

In this combination, the presence of a primary AR that leads to LV dilation and thus secondary MR, is the most common phenotype. This condition has been reported to occur in up to 45% of cases of AR, and is also considered a sign of a more advanced stage of AR [29]. Less frequently, the coexistence of MR and AR may be due to rheumatic disease, myxomatous degeneration with prolapse connective tissue disease leading to annulus dilation of both valves, or in patients with acute endocarditis [30].

The subsequent volume overload is typically badly tolerated and patients show progressive LV dilation and dysfunction.

Upon echocardiographic evaluation, the AR-related LV filling occurring before the forward flow across the mitral valve contributes to a delayed mitral valve opening and consequently to a longer isovolumetric relaxation time. Moreover, LV diastolic pressure rapidly increases because of simultaneous filling by the AR and the flow through the mitral valve. As a result, there may be a reduction in the pulmonary acceleration time to less than 100 ms, and an elevation in systolic pulmonary artery pressure can manifest even in the initial stages. Likewise, the shorter transmitral E-wave acceleration and deceleration, along with a decrease in the velocity of the A-wave, are indicative of a significant hemodynamic effect of the MR and AR [31].

Regarding the severity assessment, there are no specific recommendations to date. In some cases of AR and, more frequently, MR, multiple jets are found because of the plane of the echocardiographic beam displaying a non-circular EROA, which is quite common in secondary regurgitant lesions or bicuspid aortic valves. When multiple jets are present, the mean value of the VC on the four- and two-chamber views can be valid even though no validated cutoffs have been established so far [17, 32]. As far as quantitative methods are concerned, the PISA method is preferred, despite its known limitations [33]. Moreover, since the regurgitations are sequential in the cardiac cycle, the addition of the two single RVs would define the total RV, thereby potentially leading to determining the resulting hemodynamic effect of an AR-MR combination when each of the single lesions appear not significant [31]. Eventually, Hagendorff *et al*. [31] suggest that a significant hemodynamic impact is reasonable for a Qp/Qs ratio ≤0.74, thus having an aortic regurgitant fraction ≥35%.

### 3.4 Aortic Regurgitation and Mitral Stenosis

The coexistence of MS and AR is responsible for creating opposed loading conditions and therefore the LV does not dilate and the stroke volume does not increase as much as they usually do in case of the isolated presence of AR [13].

**Fig. 2.**
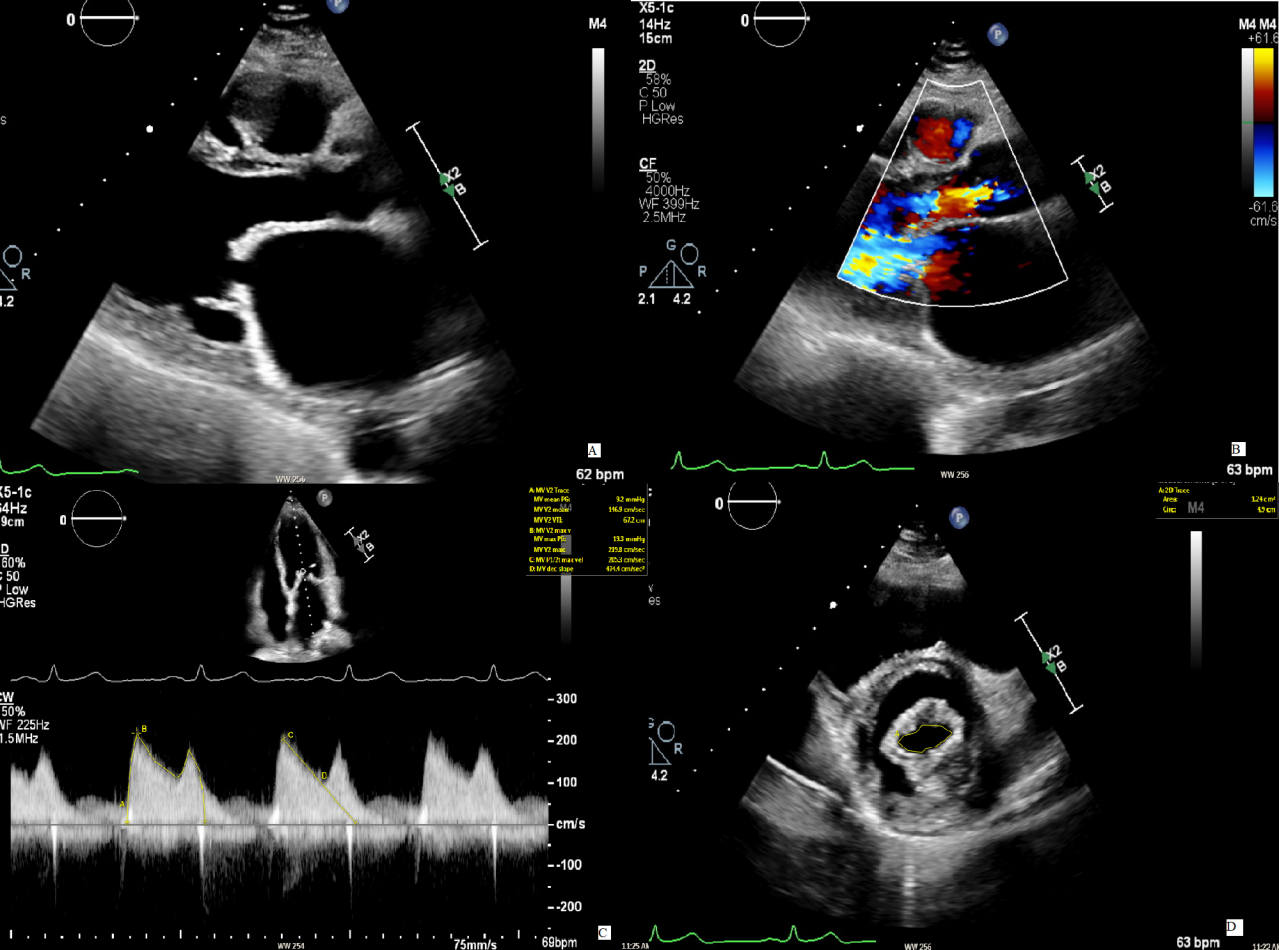
**Mitral stenosis in concomitant mitral regurgitation**. (A) The image displays a case of rheumatic mitral stenosis, recognizable by the typical “hockey stick” morphology of AMVL in diastole. (B) A concomitant moderate AR is present. (C) To evaluate the severity of the MS, the continuous wave signal over the mitral valve is used to measure the PHT from with the MVA is then calculated. The resulting value (1.7 ${\mathrm{cm}}^{2}$) accounts for a mild stenosis. (D) Nonetheless, the planimetry of the mitral valve reveals an area of 1.2 ${\mathrm{cm}}^{2}$ as for a clinically significant stenosis. The discrepancy is explained by a shorter PHT because of the rapid left ventricular filling due to the presence of AR. AMVL, anterior mitral valve leaflet; AR, aortic regurgitation; MS, mitral stenosis; MVA, mitral valve area; PHT, pressure half time.

However, the correct assessment of MS is usually a major challenge. Indeed, the presence of AR increases LV diastolic pressure causing a reduction in pressure half-time (PHT) and thus an overestimation of MVA, as shown in Fig. 2 [34, 35].

Similarly, the continuity equation for MVA calculation is also unreliable, because of the different flows over the two valves in the case of AR.

Thus, in the setting of an AR, we recommend using planimetry for MVA whenever possible. The PISA method remains more accurate than the PHT in assessing MVA, being a more reliable alternative in patients with combined AR and MS and when planimetry images are unsuitable for anatomic evaluation [36, 37].

### 3.5 Tricuspid Valve Disease and Left-Sided Valve Diseases

Rheumatic heart disease can affect the aortic, mitral and tricuspid valves at the same time, and, although at a very high risk, the correction of all the lesions is of utmost importance. However, in Western countries, TR is much more frequently secondary to a left-sided valve lesion [38].

The degree of TR is strongly influenced by modifications in the cardiovascular loading conditions. In fact, the absence of TR at a certain moment in the history of a left-sided heart valve disease, does not guarantee that TR will not develop long term. This is why echocardiography is vital for evaluating factors like the measure of the tricuspid annulus, dilation of the right chambers, right ventricular dysfunction, and the estimation of pulmonary artery pressures. These assessments are not only valuable to assess the severity of secondary TR but also to determine whether it is advisable to address surgery on the tricuspid valve in conjunction with left-sided valve surgery [10]. In particular, a combined intervention on the tricuspid valve is suggested when the end-diastolic annular dimension exceeds 40 mm (or 21 ${\mathrm{mm/m}}^{2}$).

Nowadays, the most common valvular combination is AS and TR, as moderate or more TR have been documented in 11% to 27% of patients undergoing TAVR in observational registries [39].

Even though the presence of AS does not affect the assessment of the severity of TR, pulmonary hypertension related to the presence of AS can worsen or even determine some grade of TR. Moreover, in the case of chronic and severe TR, a low flow pattern may develop, which can render the aortic gradients alone unreliable for estimating the severity of AS, tending to be underestimated [33, 40].

Since moderate-to-severe TR is an independent predictor of mortality and reoperation for secondary TR is characterized by an operative mortality risk of 10 to 25%, patients with even mild-to-moderate secondary TR with signs of right-sided heart failure or annular dilatation are generally recommended to undergo tricuspid valve surgery at the time of correction of the left-sided valve lesion [41, 42]. Nowadays, percutaneous solutions are available to successfully treat secondary TR, at the time of the other percutaneous intervention on the aortic or mitral valve, or as a staged procedure [43].

## 4. Advanced Echocardiography and Multimodality Imaging

Multimodality imaging for the diagnosis of VHD has been extensively studied and applied in the field of single valvular lesions. However, advanced echocardiography and multimodality imaging can be applied also to MVD [44].

*Transesophageal echocardiography* (TEE), although not routinely performed, can be useful in cases of diagnostic uncertainty regarding the severity of a lesion, since the advice is to prefer direct planimetry in assessing the area of stenotic valves in case of MVD. Real-time three-dimensional (3D) TEE using multiplanar reconstruction can be valuable to measure MVA in rheumatic MS when concomitant AS or AR makes Doppler measurements less reliable [45]. Moreover, in certain cases of degenerative calcified MS, the application of real-time 3D echocardiography with color-defined planimetry has proven to be beneficial [46]. Eventually, TTE intraprocedural guidance is of critical importance in valve-in-valve procedures, to overcome the challenges of such a complex intervention, especially when venous access with a subsequent transeptal approach is chosen, for instance in the case of mitral prosthesis degeneration [47].

*Stress echocardiography* is advised when symptoms cannot be explained by the resting hemodynamics and echocardiographic findings [48]. Indeed, when non-severe lesions are involved, exercise can worsen the hemodynamic consequences of the dominant lesion and end up producing symptoms. The evaluation of more than one valve during exercise is doable thanks to the combination of Doppler imaging and color flow. For example, an increase of pulmonary artery pressure >60 mmHg might help in setting the indication and consequently the timing for valve correction, as it could reflect a significant hemodynamic effect of non-severe yet combined valvular lesions [49].

*Dobutamine stress echocardiography* can be proposed for individuals with low flow-low gradient AS in case of concomitant MS or MR, to rule out pseudo-severe AS. However, when significant MR or MS are present, dobutamine may not be able to produce the necessary increase in flow, since the low flow is secondary to the valvulopathy and not to the systolic function, thereby not allowing the confirmation of AS severity [50].

*Speckle-tracking echocardiography *is known as one of the best modalities for the diagnosis and prognosis of valvular lesions, thanks to its ability to detect subclinical myocardial dysfunction before the onset of a reduction in LV ejection fraction [51]. Studies on single valvular heart disease have already shown the prognostic impact of strain analysis in such patients [52, 53, 54, 55]. Similarly, we could use speckle-tracking echocardiography to determine the correct intervention time in the setting of MVD. To date, data on the diagnostic and prognostic utility of strain analysis in MVD are lacking. A study on 72 patients showed that LV strain parameters were not altered, but RV strain parameters were mildly reduced, suggesting an overload of the RV due to the presence of combined left-sided valvulopathies [56]. Further studies are needed to investigate the role of deformation index analysis in patients with MVD.

**Fig. 3.**
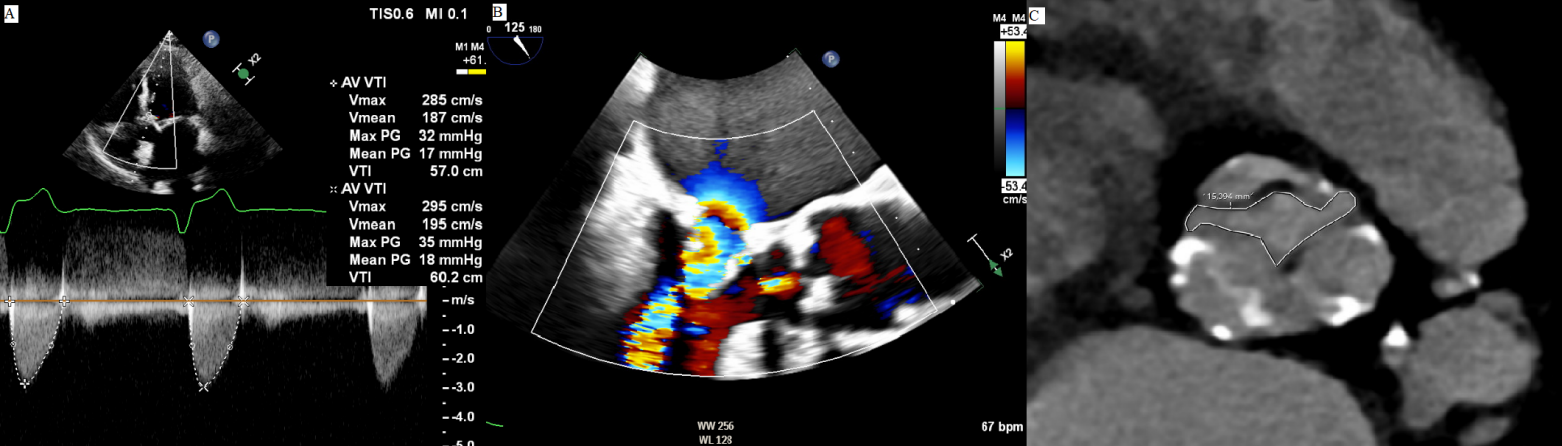
**Aortic valve planimetry on cardiac computer tomography**. (A) On transthoracic echocardiography aortic stenosis is diagnosed, with gradients across the valve compatible with a mild disease. (B) However, mitral stenosis is coexistent, thus creating a low flow state that makes the measured gradients over the aortic valve unreliable. (C) A direct planimetry obtained by a cardiac CT reveals an aortic valve area of 1.1 ${\mathrm{cm}}^{2}$. CT, computed tomography; AV, aortic valve; VTI, velocity time integral; PG, pressure gradient; WW, window width; WL, window level.

Given the challenges of MVD and especially when echocardiography fails in giving conclusive results, *multi-slice non-contrast electrocardiogram-gated computed tomography* (CT) can be used as a complementary tool. For instance, the CT-derived aortic valve calcium scoring is a reliable technique to quantify the burden of calcification on the aortic valve and it is a validated parameter of AS severity. Cutoffs for severe AS are >2000 AU in men and >1200 AU in women [57]. The calcium score offers the significant advantage of not being dependent on the patient’s hemodynamic status. This is particularly important in low-flow situations, which is often the case in the presence of MVD. Additionally, the calcium score impacts the prognosis, predicting disease progression and patient survival, irrespective of clinical and Doppler echocardiographic information [58, 59]. Furthermore, when measuring the anatomic AVA using planimetry on contrast-enhanced scans, there is a reasonable correlation with measurements obtained through the continuity equation in echocardiography, even though CT tends to systematically overestimate the AVA (Fig. 3) [60]. CT is also essential in procedural planning, especially for patients at high surgical risk, who could therefore benefit from a transcatheter approach. In this setting, CT can aid the assessment of valve characteristics that make it eligible for a full interventional solution or individuate contraindications that can instead suggest the need for surgery or a hybrid approach [61]. For instance, visualization of calcium can be limited on echocardiography, yet the presence and extent of calcification on the mitral valve are relevant in the context of the edge-to-edge technique and can be easily assessed with CT. Moreover, a CT-derived estimation of the risk of LV outflow tract obstruction is mandatory before declaring eligibility for mitral valve transcatheter replacement. As for the aortic valve, the role of CT is not limited to studying the valve features but extends to the evaluation of potential access routes beyond the classical transfemoral approach [62].

**Fig. 4.**
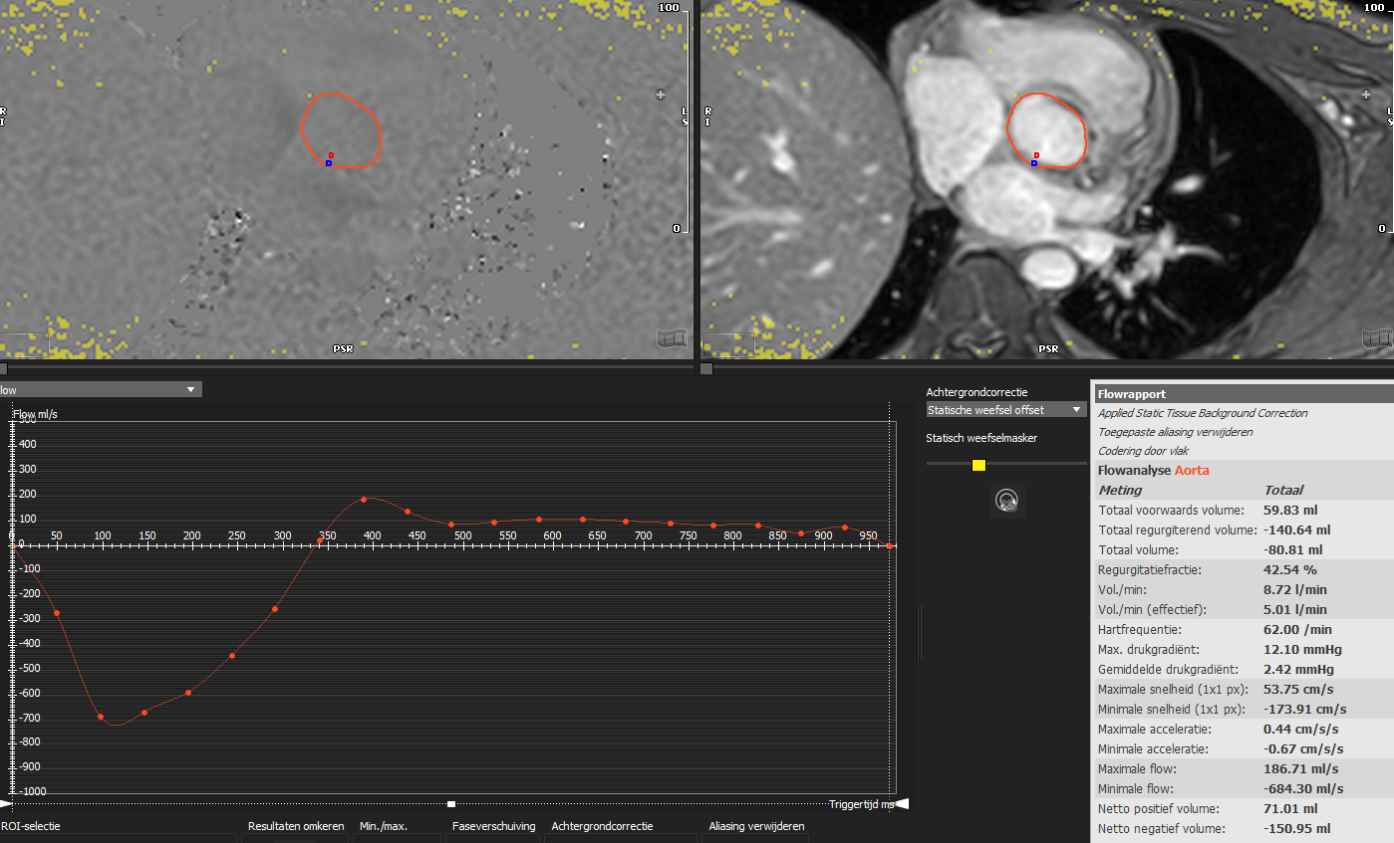
**Aortic flow quantification on cardiac magnetic resonance phase contrast imaging**. Flow quantification in the ascending aorta allows for quantification of forward volume, regurgitant volume and regurgitant fraction irrespective of the presence of other combined valvulopathies. In the image, a patient with both aortic and mitral regurgitation and left ventricle dilation underwent cardiac magnetic resonance in order to determine the severity of the main lesion, being the aortic regurgitation. The analysis revealed a regurgitant fraction of 42%, compatible with a significant aortic regurgitation.

Despite the little evidence so far, *cardiac magnetic resonance* (CMR) looks full of promise in the field of MVD. Indeed, the grading of regurgitant lesions is accurate and doesn’t suffer from the known limitations encountered in the echocardiographic assessment. The use of phase-contrast CMR with quantification of the flow in the aorta or pulmonary artery (as depicted in Fig. 4) is the recommended approach for determining the regurgitant volume and fraction. Conversely, the calculation of the regurgitant volume as the difference between the left and right stroke volumes obtained in cine-sequences can be deceptive and may not provide accurate results when more than one valvular lesion is present [63]. Even though current consensus suggests an RF of 50% as a cutoff for severe MR and AR on CMR [32], data show that an RF of 34% or more for AR and 41% or more for MR has a significant impact on prognosis [64, 65].

For individuals with AS, it is possible to obtain peak velocity and mean pressure gradient using phase-contrast sequences. However, these measurements often appear lower than those derived from Doppler analysis because of partial volume averaging within VC [63]. Similarly, CMR can assess both functional and anatomic AVA, even though at the moment they mainly remain in the realm of research. Specifically, steady-state free precession sequences offer outstanding contrast between the blood and the myocardium, along with a high signal-to-noise ratio, which allows for the measurement of the anatomic AVA. In a study by Woldendorp *et al*. [66], they found that CMR-derived anatomic AVA displayed high accuracy when compared to TEE, despite potential challenges in measurement due to jet turbulence and calcifications on the valve leaflets. The calculation of functional AVA can be achieved through phase-contrast velocity mapping, where the velocity-time integral in both the LV outflow tract and the aortic valve orifice is measured. However, there is limited knowledge regarding how well this measurement aligns with other diagnostic methods [67, 68]. Moreover, CMR is known as the most reliable technique for the quantification of ventricular volumes, thicknesses and ejection fraction, thus giving important data regarding the volume and pressure overload in MVD, that can modify the timing of invasive treatment. Eventually, CMR offers the possibility of myocardial tissue characterization, both as replacement fibrosis and diffuse fibrosis, represented by late gadolinium enhancement and extracellular volume, respectively. Recent studies point out how extracellular volume could emerge as an interesting technique to outline the presence of myocardial overload, in advance of the onset of late gadolinium enhancement, thus potentially refining the optimal timing for intervention [69, 70]. However, differently from echocardiographic criteria that have been validated against clinical outcomes, such data are not available yet for CMR parameters and further studies need to prove their diagnostic and prognostic value.

## 5. Other Resources for the Non-Invasive Assessment of Multivalvular Heart Disease

In patients with heart failure, brain natriuretic peptide (BNP) levels predict exercise performance and prognosis and, in patients with single valve disease, an increase of BNP levels has been shown to correlate with the severity of valve lesion and LV dimensions [71, 72]. NT-proBNP level was demonstrated to be a dominant predictor of peak oxygen consumption at the cardiopulmonary exercise testing, while traditional markers of valve disease severity as ejection fraction, fractional shortening, and diastolic dysfunction were only moderately correlated with the exercise capacity [73]. Even though cutoffs are lacking, these results suggest that in the setting of moderate to severe MVD additional information on functional capacity and hemodynamic effect can be provided by the serial testing of natriuretic peptides, especially in the asymptomatic or vaguely symptomatic patients, on top of clinical evaluation and echocardiography.

As for cardiopulmonary exercise testing, MVD patients may have a functional capacity impairment, which can be difficult to detect from their clinical presentation or from a yet accurate anamnesis, because patients tend to reduce their physical activity and become deconditioned. This represents an obstacle to an exhaustive assessment, especially considering the fact that current indications for intervention often require the presence of symptoms, usually as dyspnea [10]. In a study by Bissessor *et al*. [74] patients with MVD achieved lower peak oxygen consumption in comparison to controls, even when asymptomatic. Moreover, there was no significant difference in the echocardiographic severity of the valve lesions between different New York Heart Association (NYHA) classes, yet different exercise performance, and the peak oxygen consumption was a predictor of poor outcome.

These findings support the use of natriuretic peptides sampling and cardiopulmonary exercise testing next to imaging assessment in risk stratification and thus in the decision-making process of the timing of intervention.

## 6. Future Perspective

Trials are ongoing with the aim to better study and define MVD. Among them, the multicentric *Aortic+Mitral TRAnsCatheter (AMTRAC) Valve Registry* is studying the characteristics and outcomes of patients undergoing TAVR with a concomitant MR (ClinicalTrials.gov Identifier: NCT04031274). Aims include a better understanding of the predictors for MR regression following isolated TAVR and consequently estimating the fraction of patients who will be suitable for a transcatheter intervention on the mitral valve after TAVR. Moreover, the centers will investigate the outcomes of patients with significant MR post-TAVI who received mitral valve intervention, compared to those left for medical management.

Similarly, the *MITAVI *trial is still recruiting patients with the aim to determine if the persistence of moderate to severe MR after TAVR can benefit from an additional treatment of this valve disease as well (ClinicalTrials.gov Identifier: NCT04009434).

Also recruiting patients is the *TIAMAR* study, to investigate the safety and efficacy of early (within 3 months) versus deferred aortic valve replacement in patients with moderate AS combined with moderate MR (ClinicalTrials.gov Identifier: NCT05310461).

## 7. Conclusions

Despite the remarkable prevalence of MVD, current guidelines on diagnosis and management of VHD mostly focus on single valve diseases and, when MVD is addressed, the majority of indications are reserved for treatment of concomitant valve lesions in patients with a primary indication to surgery for another valve [10]. However, the combination of multiple non-severe lesions may result in hemodynamically severe consequences, symptoms and systolic or diastolic dysfunction.

Clinicians must be aware of the wide range of clinical scenarios associated with MVD. At the same time, early management of these patients is of key importance to improve their prognosis before the occurrence of symptoms and LV damage. Therefore, an extensive knowledge of echocardiographic pitfalls is fundamental while evaluating these patients, thus making a multimodality assessment of MVD of paramount importance. Further studies are needed to provide imaging cardiologists with a multimodality assessment of MVD and to guide valve teams in treatment decision-making for these complex clinical cases.

## Abbreviations

3D, three-dimensional; AR, aortic regurgitation; AS, aortic stenosis; AVA, aortic valve area; CMR, cardiac magnetic resonance; CT, computed tomography; EROA, effective regurgitant orifice area; LV, left ventricle; MR, mitral regurgitation; MS, mitral stenosis; MVA, mitral valve area; MVD, multivalvular heart disease; PHT, pressure half-time; PISA, proximal isovelocity surface area; RF, regurgitant fraction; RV, regurgitant volume; TAVI, transcatheter aortic valve implantation; TEE, transesophageal echocardiography; TR, tricuspid regurgitation; VC, vena contracta; VHD, valvular heart disease.
